# Supplementary material for: Central amygdala circuitry modulates nociceptive processing through differential hierarchical interaction with affective network dynamics
Source: Commun Biol. 2021 Jun 14;4:732. doi: 10.1038/s42003-021-02262-3 (PMC8203648; doi:10.1038/s42003-021-02262-3)
Supplement: Supplementary file 2 — Supplementary Material [file 42003_2021_2262_MOESM2_ESM.pdf]

Supplementary Material for

Central amygdala circuitry modulates nociceptive processing through differential hierarchical interaction with affective network dynamics

Isabel Wank[1$,] Pinelopi Pliota[2$], Sylvia Badurek[3], Klaus Kraitsy[3], Joanna Kaczanowska[2], Johannes Griessner[2], Silke Kreitz[1], Andreas Hess[1#], Wulf Haubensak[2#*]

[1]Institute of Experimental and Clinical Pharmacology and Toxicology, Friedrich-Alexander University Erlangen-Nuremberg, Fahrstrasse 17, 91054 Erlangen, Germany

[2]Research Institute of Molecular Pathology (IMP), Vienna Biocenter (VBC), Dr. Bohr-Gasse 7, 1030 Vienna, Austria

[3]Preclinical Phenotyping Facility, Vienna Biocenter Core Facilities GmbH (VBCF), Dr. Bohr Gasse 3, 1030, Vienna, Austria

[$]These authors contributed equally to this manuscript.

[#]These authors contributed jointly supervised this work.

[*]To whom correspondence should be addressed: wulf.haubensak@imp.ac.at

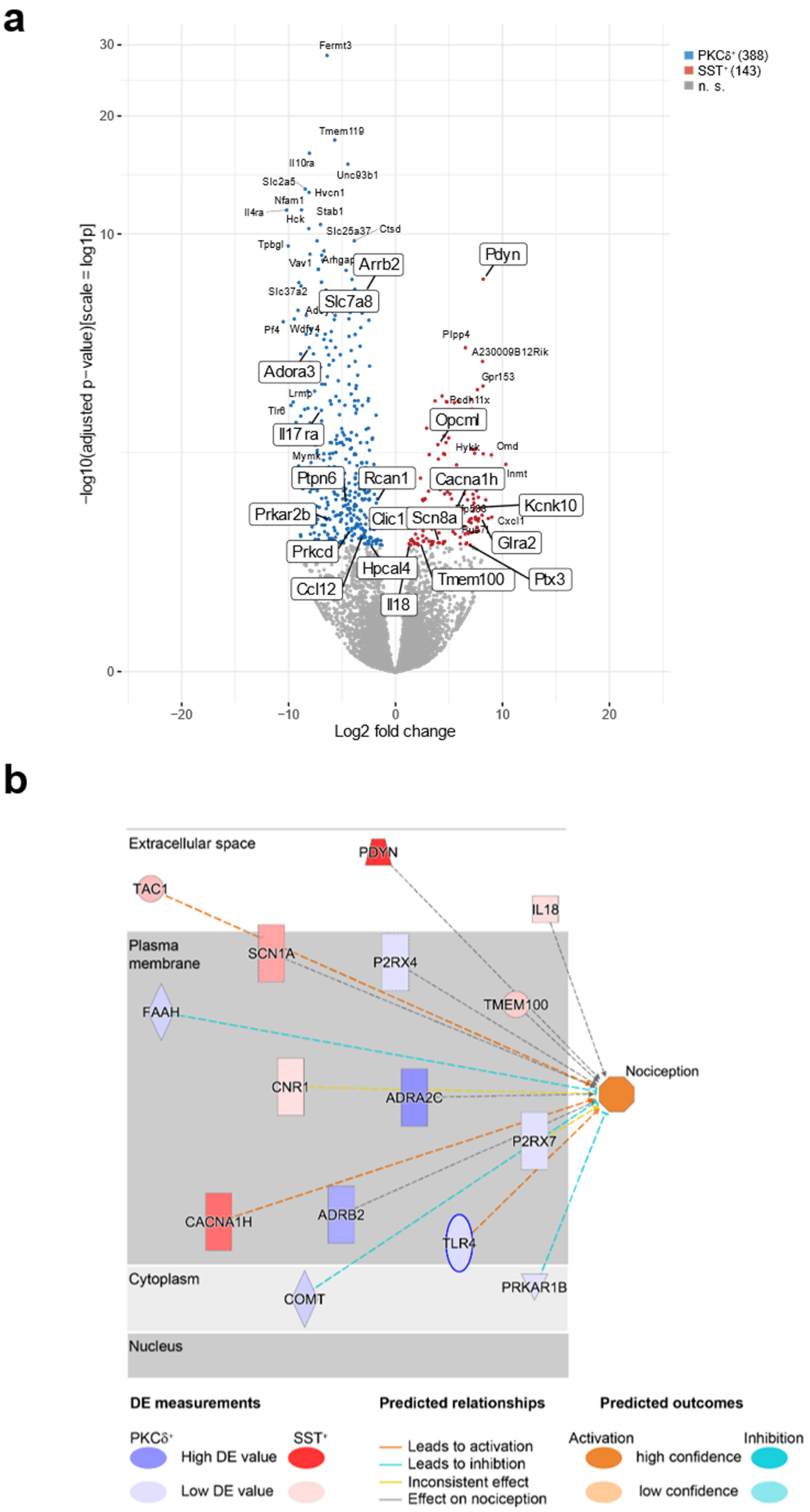
a
Fermt3
Tmem119
Il10ra
Unc93b1
Slc2a5
Hvcn1
Nfam1
Il4ra
Hck
Stab1
Slc25a37
Ctsd
Tpbgl
Vav1
Arrb2
Pdyn
Slc37a2
Slc7a8
Pf4
Plpp4
A230009B12Rik
Adora3
Gpr153
Lrmp
Tlr6
Il17ra
Opcml
Omd
Inmt
Ptpn6
Rcan1
Cacna1h
Kcnk10
Prkar2b
Clic1
Scn8a
Cxcl1
Glra2
Prkcd
Hpcal4
Tmem100
Ptx3
Ccl12
Il18
PKCδ⁺ (388)
SST⁺ (143)
n. s.
−log10(adjusted p−value)[scale = log1p]
Log2 fold change
b
Extracellular space
PDYN
TAC1
IL18
Plasma membrane
SCN1A
P2RX4
TMEM100
FAAH
Nociception
CNR1
ADRA2C
P2RX7
CACNA1H
ADRB2
TLR4
Cytoplasm
COMT
PRKAR1B
Nucleus
DE measurements
PKCδ⁺
SST⁺
High DE value
Low DE value
Predicted relationships
Leads to activation
Leads to inhibition
Inconsistent effect
Effect on nociception
Predicted outcomes
Activation
Inhibition
high confidence
low confidence

Supplementary Figure 1: Nociception-related genes expressed in two CEl cell populations. **a** Expression of nociception-related genes in CEl PKCδ$^+$ (blue) or SST$^+$ (red) neuronal populations. Differential expression (DE) values of expressed genes (TPM >1 in any PKCδ$^+$ (blue) or SST$^+$ (red) sample) are averaged per gene across 4 sequencing replicates per population with an FDR of 0.1. The total number of DE genes upregulated in either population are given in brackets. Pain related genes (Supplementary Table 1, DE Pain gene set) are boxed. **b** Ingenuity Pathway Analysis 'Diseases or functions' annotation of the pain-gene set (Supplementary Table 1, IPA Pain gene set; Fig. 1). The pain gene set associated strongly with 'nociception' ($p=4,6 \times 10^{-19}$). The DE values overall differential effects of pain gene expression in SST$^+$ (red) compared to PKCδ$^+$ neurons (blue) with a predicted main gene network effect in increasing nociception (orange) (note that for FAAT the IPA prediction differs from its canonical analgesic effects and that not all genes reach significance threshold for individual DE values).

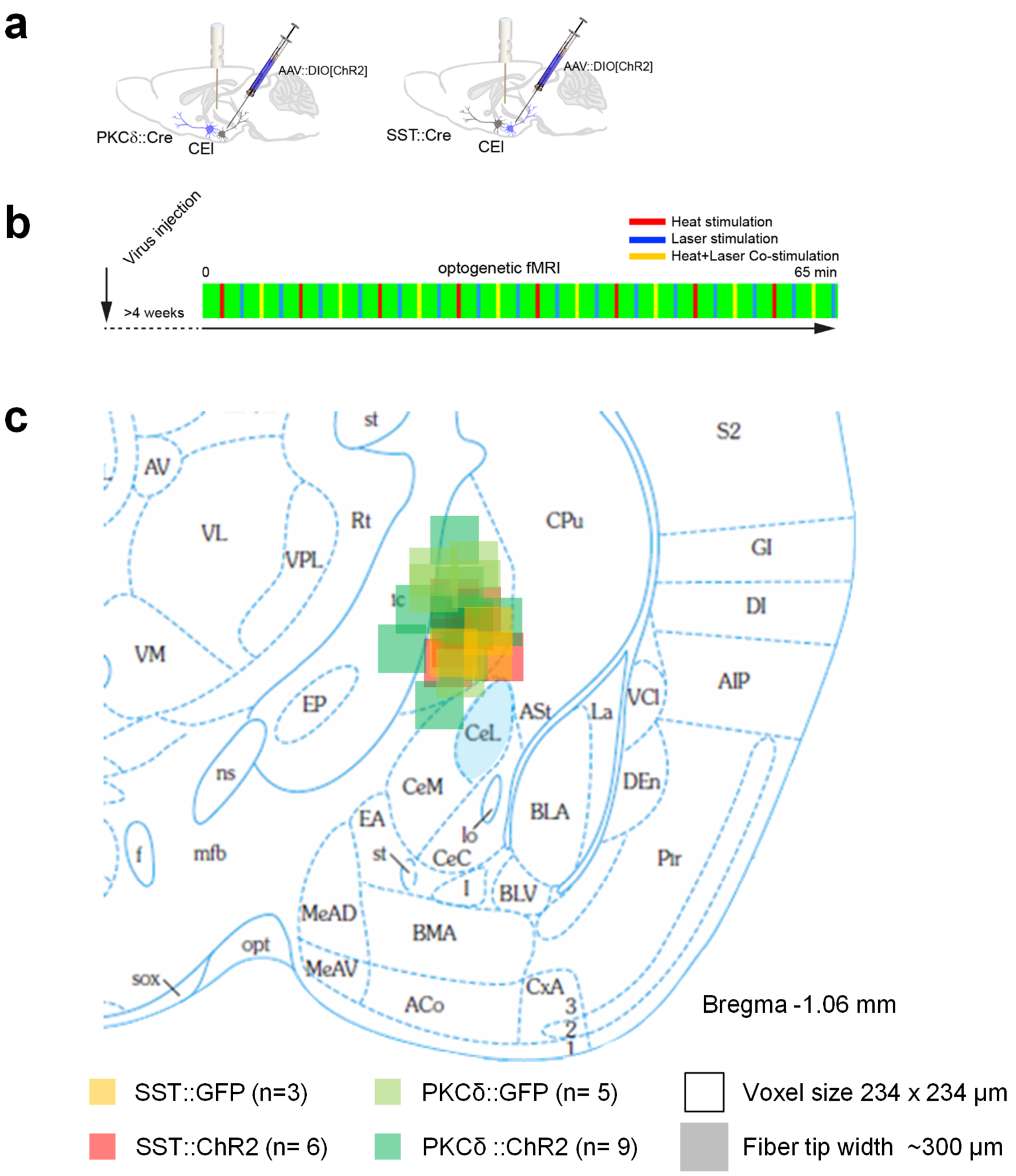

Supplementary Figure 2: Optogenetic fMRI experimental design.
a Viral targeting of ChR2 expression to PKCδ^{+} and SST^{+} neurons in CEl. **b** Optogenetic fMRI stimulation paradigm. **c** Stereotaxic placement of optogenetic fibers: positions of stereotaxic surgeries with combined viral injection and fiber implantation of the experimental cohort was verified from anatomical MRI scans. The color-coded squares indicate the anatomical position of the fiber tips (drawing taken from[1]). One animal with incorrect stereotaxic targeting was excluded from the analysis (not shown).

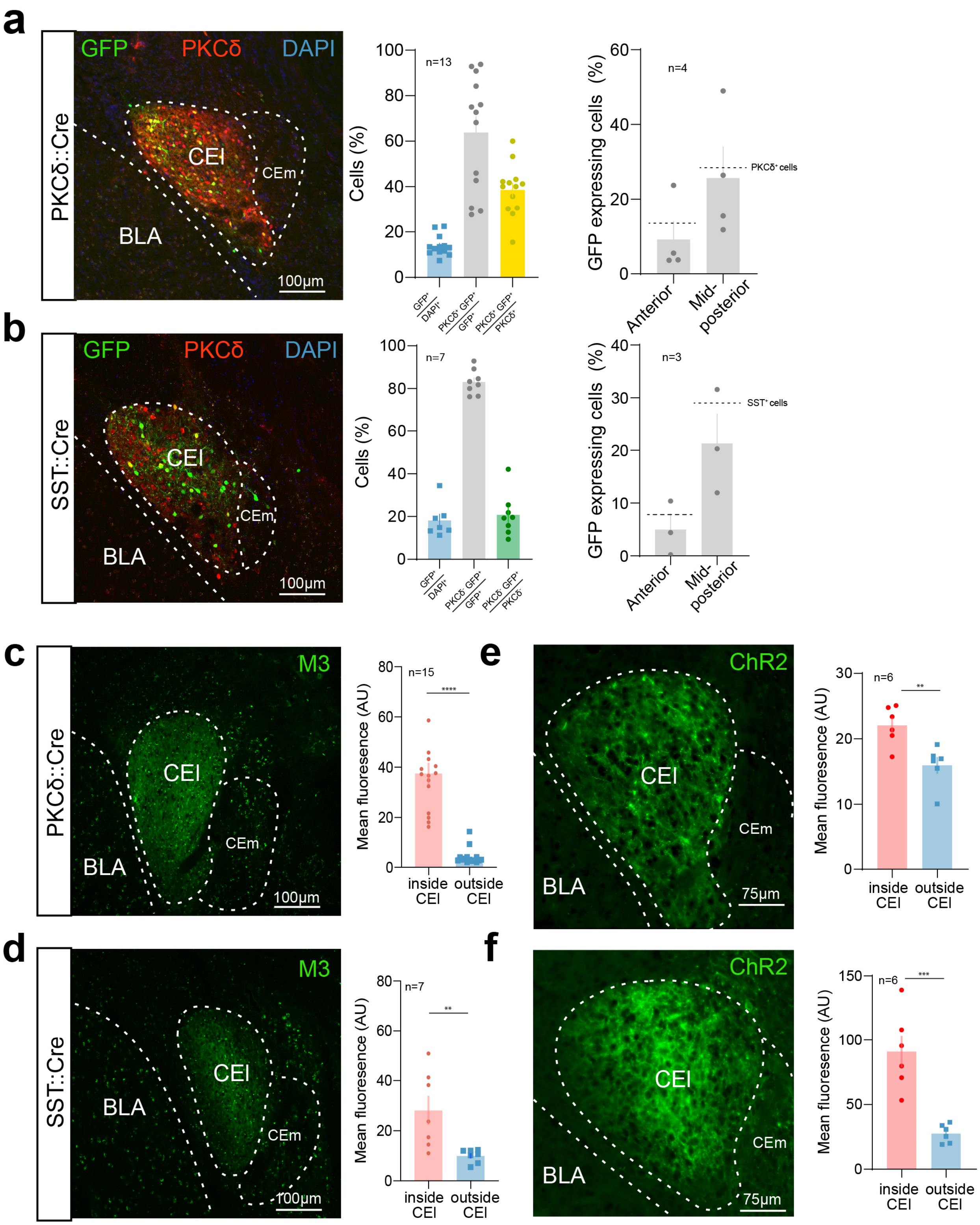
a
PKCδ::Cre
GFP
PKCδ
DAPI
CEl
CEm
BLA
100µm
n=13
Cells (%)
n=4
GFP expressing cells (%)
PKCδ⁺ cells
Anterior
Mid-posterior
b
SST::Cre
GFP
PKCδ
DAPI
CEl
CEm
BLA
100µm
n=7
Cells (%)
n=3
GFP expressing cells (%)
SST⁺ cells
Anterior
Mid-posterior
c
PKCδ::Cre
M3
CEl
CEm
BLA
100µm
n=15
Mean fluorescence (AU)
inside CEl
outside CEl
d
SST::Cre
M3
CEl
CEm
BLA
100µm
n=7
Mean fluorescence (AU)
inside CEl
outside CEl
e
ChR2
CEl
CEm
BLA
75µm
n=6
Mean fluorescence (AU)
inside CEl
outside CEl
f
ChR2
CEl
CEm
BLA
75µm
n=6
Mean fluorescence (AU)
inside CEl
outside CEl

Supplementary Figure 3: Viral targeting of CEl circuitry.

a,**b** Cre-recombination allows for specific and efficient viral targeting of PKCδ$^+$ and SST$^+$ cells in CEl[2]. Exemplary pictures of PKCδ::Cre (**a**, left) and SST::Cre (**b**, left) transgenic animals injected with cre-dependent AAV-expressing GFP. Cellular specificity and efficiency of viral injection (middle). GFP expression in both anterior and mid/posterior subdomains of CEl in PKCδ::Cre and SST::Cre animals (right). SST$^+$ cells were also distributed along anterior-posterior axis. In both cases the PKCδ$^+$ and SST$^+$ cells followed the distribution of their respective markers (dotted lines in right-sided bar graphs: PKCδ marker expression data, values computed from n=4 animals; SST marker expression data, values taken from McCullough *et al*[3]. This suggests that manipulations are not specific for either anterior or mid-posterior functional subpopulations of PKCδ$^+$ or SST$^+$ cells[4]. **c-f**, DREADDs-hM3Gq (**c**,**d**) or ChR2 (**e**,**f**) from the experimental cohorts used for behavioral experiments in Supplementary Figure 6 and from an animal cohort injected under the same conditions as animals used in fMRI Figs. 2-3 and Supplementary Figs. 4 and 7 were also reliably expressed in CE neuronal populations (C, t=7.651, $p < 0.0001$; D, t=3.090, P=0.0094; E, t=3,474 P=0.0060; F, t=5.12, P=0.0005). Note that due to the targeting of DREADDs-hM3Gq and ChR2 to neurites, quantification was performed on regional (mean fluorescence), and not cellular basis as in **a**,**b**. This notwithstanding, DREADDs-hM3Gq and ChR2 can be expected to be cell type specific as observed for GFP (**a**,**b**) and described previously[5]. Bars are mean ± SEM.

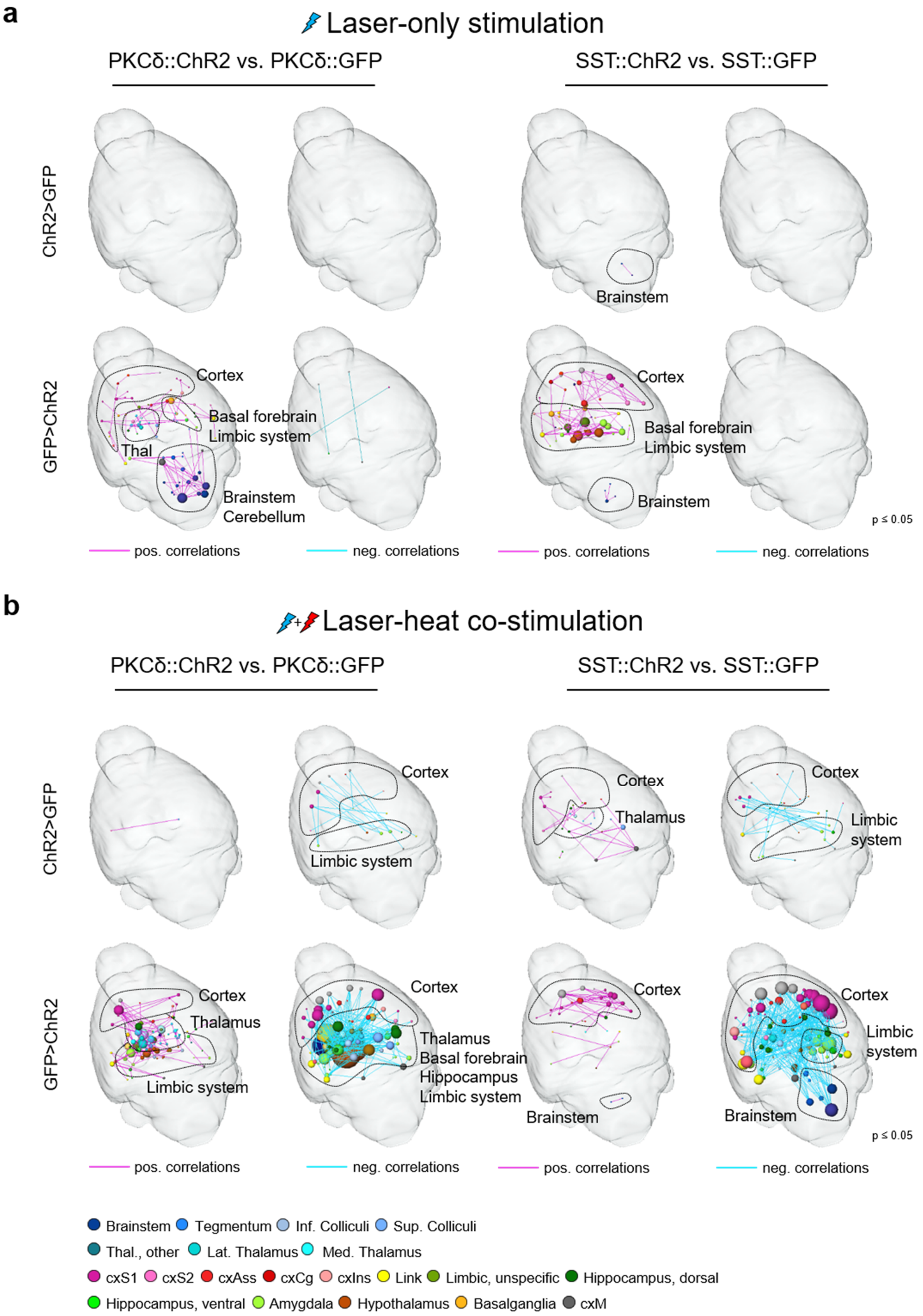
a
Laser-only stimulation
PKCδ::ChR2 vs. PKCδ::GFP
SST::ChR2 vs. SST::GFP
ChR2>GFP
GFP>ChR2
Brainstem
Cortex
Basal forebrain
Limbic system
Thal
Brainstem
Cerebellum
Cortex
Basal forebrain
Limbic system
Brainstem
p ≤ 0.05
pos. correlations
neg. correlations
pos. correlations
neg. correlations
b
Laser-heat co-stimulation
PKCδ::ChR2 vs. PKCδ::GFP
SST::ChR2 vs. SST::GFP
ChR2>GFP
GFP>ChR2
Cortex
Limbic system
Cortex
Thalamus
Cortex
Limbic system
Cortex
Thalamus
Limbic system
Cortex
Thalamus
Basal forebrain
Hippocampus
Limbic system
Cortex
Brainstem
Cortex
Limbic system
Brainstem
p ≤ 0.05
pos. correlations
neg. correlations
pos. correlations
neg. correlations
Brainstem
Tegmentum
Inf. Colliculi
Sup. Colliculi
Thal., other
Lat. Thalamus
Med. Thalamus
cxS1
cxS2
cxAss
cxCg
cxIns
Link
Limbic, unspecific
Hippocampus, dorsal
Hippocampus, ventral
Amygdala
Hypothalamus
Basalganglia
cxM

Supplementary Figure 4: Optogenetic activation of CEl neuronal populations differentially modulated brain functional connectivity networks.

a Significant (homoscedastic two-tailed student's t-test, $p \leq 0.05$, uncorrected) differences in functional connectivity of $PKC\delta^+$ and SST^+ neuronal activation compared to their respective controls ($n_{PKC\delta::GFP} = 5$, $n_{SST::GFP} = 3$, $n_{PKC\delta::ChR2} = 9$, $n_{SST::ChR2} = 6$) during laser application.

Compared to controls, selective activation of CEl $PKC\delta^+$ neurons reduced pCorrs (see Methods) within amygdala, cerebellum and brainstem regions, with the brainstem connectivity being greatly reduced. In a second pathway, pCorrs between amygdala, the rest of the limbic system including the basal forebrain, thalamus and cortex were reduced. NCorrs were rare, unsystematic and only found for CEl $PKC\delta^+$.

Selective activation of CEl SST^+ neurons significantly reduced pCorrs within the hypothalamus, limbic system and cortex. Brainstem functional connectivity was almost normal compared to the respective GFP-controls.

b Significant differences (homoscedastic two-tailed student's t-test, $p \leq 0.05$, uncorrected) within brain networks evoked by combined laser application and heat stimulation ($n_{PKC\delta::GFP} = 5$, $n_{SST::GFP} = 3$, $n_{PKC\delta::ChR2} = 9$, $n_{SST::ChR2} = 6$). Compared to controls, CEl $PKC\delta^+$ neurons showed a clear lateralization to the side contralateral to laser application: reduced pCorrs within the limbic system (including amygdala), basal forebrain, thalamus and cortical regions, as well as reduced nCorrs within the limbic system (amygdala, hippocampus, hypothalamus), thalamus, the basal forebrain and cortex. Enhanced nCorrs were found between ipsilateral amygdala and some contralateral cortical regions.

CEl SST^+ neurons on the other hand focally reduced ipsilateral pCorrs within cortex. PCorrs were only slightly enhanced in cortex and thalamus. NCorrs were enhanced in SST^+ neurons between ipsilateral amygdala and some contralateral cortical regions, comparable to $PKC\delta^+$ neurons. Reduced nCorrs occurred in ipsilateral amygdala, cortex and brainstem.

Color-coded spheres represent different brain regions. Size of the sphere represents the degree, i.e. the number of connections this brain region has to the others. Spheres are rendered in 3D within the mouse brain (transparent isosurface) with the corresponding Paxinos coordinates.

Abbreviations: cxS1/cxS2 (primary/secondary somatosensory cortex), cxAss (association cortex), cxCg (cingulate cortex), cxIns (insular cortex), Link (ento/ectorhinal and piriform cortex), Limbic, unspecific (habenulae, septum, diagonal band), cxM (motor cortex).

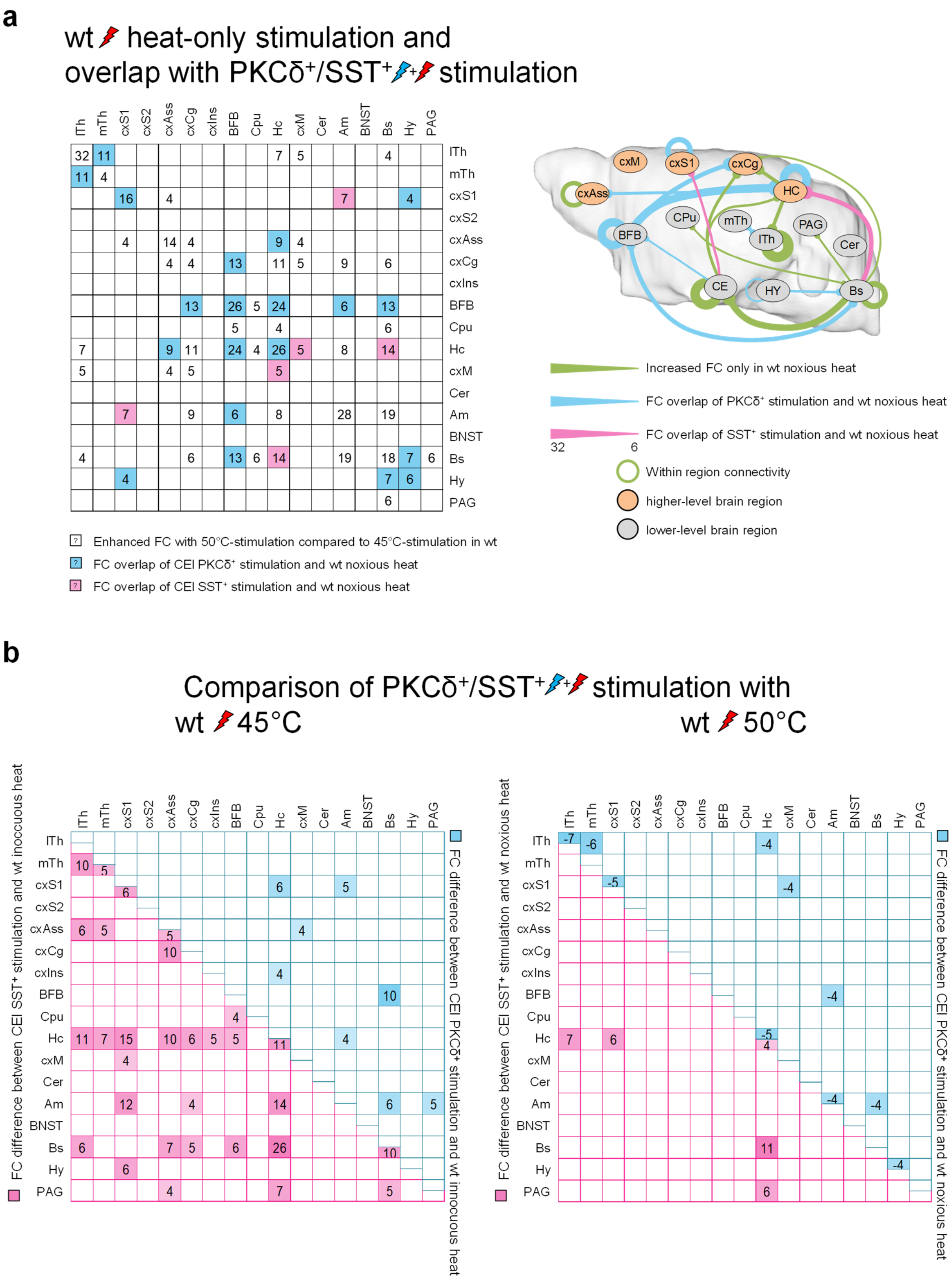
a
wt heat-only stimulation and overlap with PKCδ+/SST+ stimulation
lTh mTh cxS1 cxS2 cxAss cxCg cxIns BFB Cpu Hc cxM Cer Am BNST Bs Hy PAG
cxM cxS1 cxCg cxAss HC CPu mTh PAG BFB lTh Cer CE HY Bs
Increased FC only in wt noxious heat
FC overlap of PKCδ+ stimulation and wt noxious heat
FC overlap of SST+ stimulation and wt noxious heat
32
6
Within region connectivity
higher-level brain region
lower-level brain region
Enhanced FC with 50°C-stimulation compared to 45°C-stimulation in wt
FC overlap of CEl PKCδ+ stimulation and wt noxious heat
FC overlap of CEl SST+ stimulation and wt noxious heat
b
Comparison of PKCδ+/SST+ stimulation with
wt 45°C
wt 50°C
FC difference between CEl SST+ stimulation and wt inocuous heat
FC difference between CEl PKCδ+ stimulation and wt innocuous heat
FC difference between CEl SST+ stimulation and wt noxious heat
FC difference between CEl PKCδ+ stimulation and wt noxious heat

Supplementary Figure 5: Heat-processing connectivity matrix of wild type mice and comparison to CEl PKCδ$^+$/SST$^+$ laser + heat co-stimulation.

a Noxious heat stimulation of 50 °C in male wild type (wt, n_{WT} = 29) mice significantly enhanced (positive values) functional connectivity of lateral thalamus, cingulate cortex (cxCg), basal forebrain (BFB), hippocampus (Hc), amygdala (CE) and brainstem (Bs), compared to stimulation with innocuous 45 °C. Highly noxious information received by the Bs is distributed via CE and BFB indirectly to the key regions cxCg and Hc. The latter one is also addressed directly by Bs. Using the connectivity matrices of CEl PKCδ$^+$/SST$^+$ (Figure 3b) as mask ($n_{PKCδ::ChR2}$ = 9, $n_{SST::ChR2}$ = 6), similarities with the wt matrix could be identified (left) and displayed as a network (right). Out of 38 wt connections, 12 overlapped with CEl PKCδ$^+$ (blue), but only 3 connections overlapped with CEl SST$^+$ (pink). Even though FC is per se undirected, connections were fortified with known structural connectivity obtained by anterograde tracing studies (Allen brain atlas[6]) and displayed by schematic synapses on the target regions.

b The brain networks of CEl PKCδ$^+$/SST$^+$ laser + heat co-stimulation ($n_{PKCδ::ChR2}$ = 9, $n_{SST::ChR2}$ = 6) were directly compared to wt mice (n_{WT} = 29) stimulated with innocuous 45 °C (left) and noxious 50 °C (right). CEl PKCδ$^+$ co-stimulation (blue) showed stronger FC (positive values) compared to wt 45 °C, but weaker FC (negative values) than wt 50 °C. CEl SST$^+$ co-stimulation (pink) on the other hand, displayed significantly stronger FC than wt 45 °C, and even stronger FC than wt 50 °C (here only in hippocampal connections due to limiting the data to contain at least 4 connections).

This led us to the conclusion, that CEl PKCδ$^+$ co-stimulation reduced perception of noxious heat to a level between wt 45 and 50 °C. This effect could not be shown for SST co-stimulation.

The unit of FC used in this figure represents summed up significant changes between stimulation of wt mice with 50 °C and 45 °C, or between CEl PKCδ$^+$/SST$^+$ laser + heat co-stimulation and wt 45/50 °C, in negative and positive correlations (homoscedastic two-tailed student's t-test, $p \leq 0.05$, uncorrected). Meaning, a net FC of 6 here in this picture represents six significantly different connections (positive and/or negative Pearson *r*) that were greater for stimulation with 50 °C than with 45 °C, or, for example seven that were greater and one that was smaller for 50 °C.

Shown are changes stronger than 4 (matrices of **a** and **b**) or 6 (connections displayed as network in **b**) connections in blue for PKCδ$^+$ and pink for SST$^+$.

Abbreviations: Am (amygdala), BFB (basal forebrain including septum, diagonal band of broca, nucleus accumbens, pallidum), BNST (bed nucleus of stria terminalis), Bs (brainstem), CE (central amygdala, reflected by the respective neuronal populations, PKCδ$^+$ or SST$^+$), Cer (Cerebellum), CPu (caudate putamen), cxAss (parts of association cortex), cxCg (cingulate cortex), cxIns (insular cortex), cxM (motor cortex), cxS1 (prim. somatosensory cortex), cxS2 (secondary somatosensory cortex), Hc (hippocampus), Hy (hypothalamus), lTh (lateral thalamus), mTh (medial thalamus), PAG (periaqueductal gray).

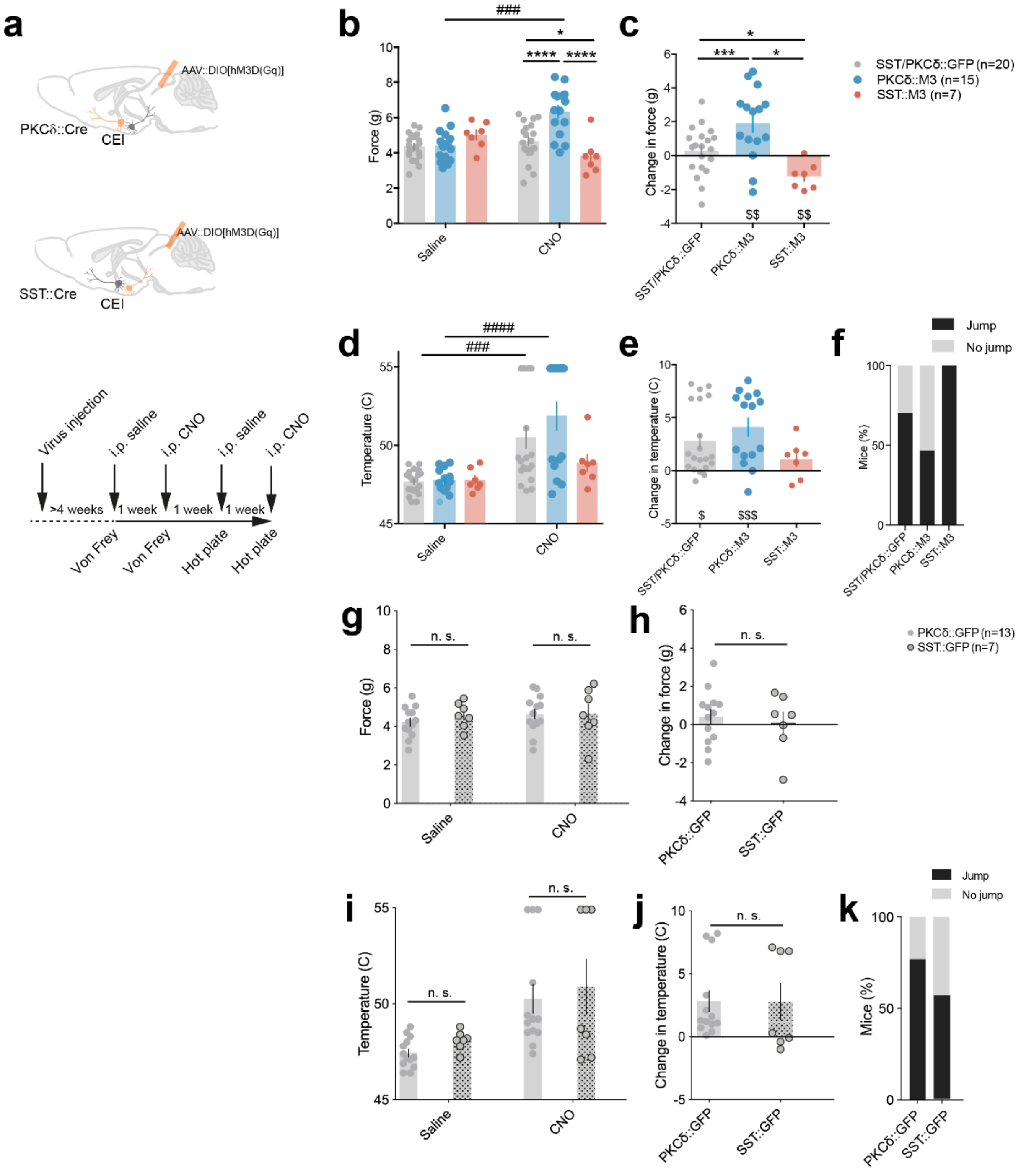
a
AAV::DIO[hM3D(Gq)]
PKCδ::Cre
CEl
AAV::DIO[hM3D(Gq)]
SST::Cre
CEl
Virus injection
i.p. saline
i.p. CNO
i.p. saline
i.p. CNO
>4 weeks
1 week
1 week
1 week
Von Frey
Von Frey
Hot plate
Hot plate
b
Force (g)
Saline
CNO
c
Change in force (g)
SST/PKCδ::GFP
PKCδ::M3
SST::M3
SST/PKCδ::GFP (n=20)
PKCδ::M3 (n=15)
SST::M3 (n=7)
d
Temperature (C)
e
Change in temperature (C)
f
Jump
No jump
Mice (%)
g
h
PKCδ::GFP (n=13)
SST::GFP (n=7)
PKCδ::GFP
SST::GFP
n. s.
i
j
k

Supplementary Figure 6: CEl PKCδ$^+$ and SST$^+$ neurons differentially modulate nociceptive behavior.

a Experimental strategy to access CEl PKCδ$^+$ and SST$^+$ neuronal impact on pain behavior. Transgenic PKCδ::Cre or SST::Cre mice were injected intra-CEl with either AAV::DIO-hM3Dq (for DREADD activation) or AAV::DIO-GFP (for control, shown in grey). Prior each von Frey trial, mice were injected i.p. with saline or 10 mg/kg clozapine-N-oxide (CNO) to pharmacogenetically activate PKCδ$^+$ (blue) or SST$^+$ (pink) neurons. Later, mice underwent a two-trial increasing temperature hot plate test. Before each trial, they received an i.p. injection of saline or CNO. **b** Pharmacogenetic activation of PKCδ$^+$ neurons with CNO (n=15) showed increased mechanical response thresholds in von Frey tests compared to PKCδ::Cre mice injected with AAV::DIO-GFP (n=13), SST::Cre mice injected with AAV::DIO-GFP (n=7) and SST::Cre mice injected with AAV::DIO M3 (n=7) after CNO injection (significance marked with *). In contrast, SST::Cre mice injected with AAV::DIO M3 had lower response thresholds under CNO treatment. Pharmacogenetic activation of PKCδ$^+$ neurons with CNO also showed increased mechanical response thresholds in von Frey test compared to saline injection (Two-way RM ANOVA, $F_{interaction}$ (2, 39)=9.430; p=0.0005; F_{group} (2, 39)=10.21; p=0.0003; $F_{treatment}$ (1, 39)=1.430; p=0.2389; Holm-Sidak *post hoc* tests, significance marked with #). **c** Compared to a theoretical mean zero, activating SST$^+$ neurons decreased (n=7, one sample t test, t(6)=4.055, p=00067) response thresholds during the CNO session, while activating PKCδ$^+$ neurons increased thresholds (n=15, one sample t test, t(14) = 3.510, p=0.0035, significance marked with $). This effect was absent in controls (n=20, t(19)=0.9180, p=0.37) and was markedly different between the two experimental groups (F (2, 39)=9.430, p=0.0005; Holm-Sidak *post hoc* test, significance marked with *). **d** Heat response thresholds in animals after PKCδ$^+$ neuron vs. SST$^+$ neuron activation trended toward higher temperatures after CNO, but not saline, injections ($F_{interaction}$ (2, 39)=2.334, p=0.1103; F_{group} (2, 39) = 2.164, p=0.1284; $F_{treatment}$ (1,39)=25.27; p<0.0001; Holm-Sidak *post hoc* tests, * indicates between groups and # between saline and CNO significance in post hoc tests). **e** Changes in heat response thresholds upon CNO vs. saline injection baselines significantly increased in controls (n=20, t(19)=3.867, p=0.001) and after activating PKCδ$^+$ neurons (n=15, t(14)=4.716, p=0.0003), but not after activating SST$^+$ neurons (n=7, t(6)=1.561, p=0.17) (significance marked with $). **f** Fraction of mice that responded with a jump reaction.

Behavior of control-groups is similar across genetic backgrounds (**g-k**). PKCδ::Cre (light grey) and SST::Cre (shaded grey) animals were injected with AAV::DIO-GFP and used as controls in behavioral and fMRI experiments. These two control-groups did not differ in their noxious responses in von Frey (**g** 2-way RM ANOVA $F_{interaction}$(1, 18)=0.2325, P=0.6355, $F_{treatment}$(1, 18)=0.4388, P=0.4848; D, t=0.002387, P=0.9812; **h** t(18)=0.4821, P=0.6355) and Hot plate (**i** 2-way RM ANOVA $F_{interaction}$(1, 18)=0.0006, P=0.9812, $F_{treatment}$(1, 18)=0.7285, P=0.4046; **j** t(18)=0.002387, P=0.9812; **k** % of jump reactions) tests and their response to CNO. These groups were therefore pooled to increase statistical power in the behavioral analysis (**a-f**).

Significance levels are given as */#/$ p < 0.05, **/##/$$ p < 0.01, ***/###/$$$ p < 0.001, ****/####/$$$$ p < 0.0001. Bars are mean ± SEM.

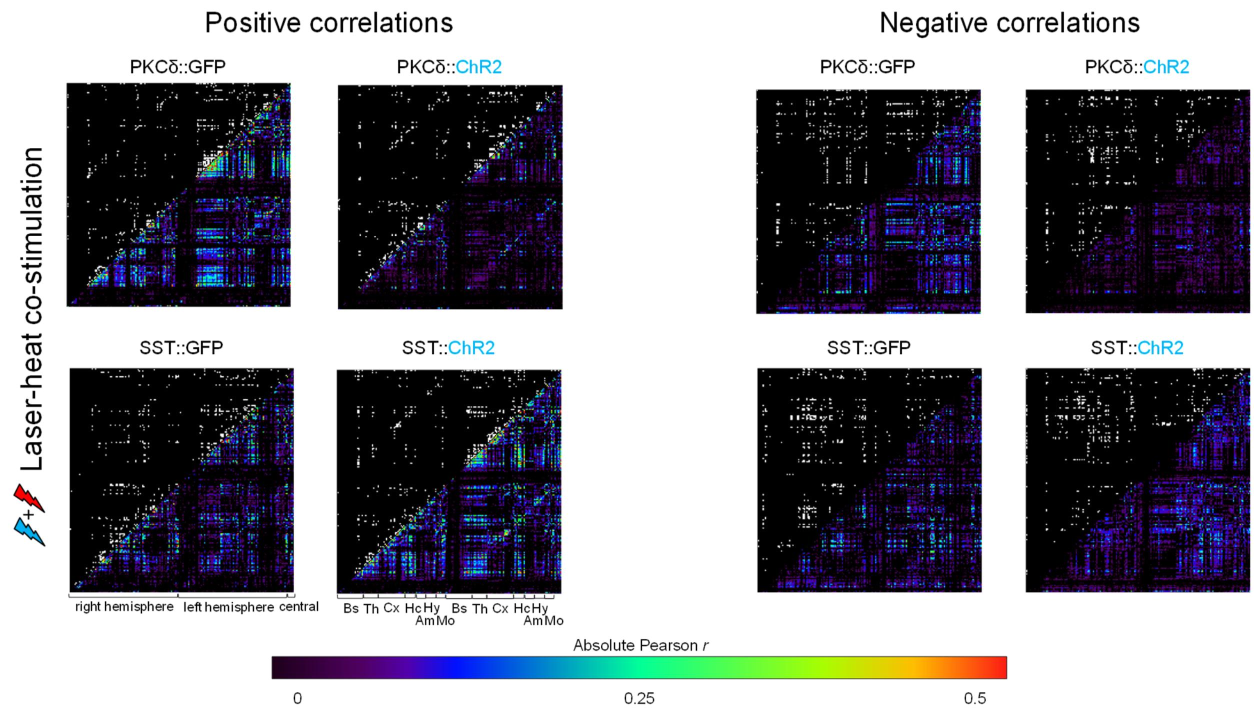

Supplementary Figure 7: Functional connectivity adjacency matrices.

Shown are the symmetric adjacency matrices of each experimental group representing positive (pCorrs) and negative (nCorrs) mean r-values of the laser-heat co-stimulation. Lower right triangles contain mean r-values for each pair of brain regions. Upper left triangles show the binary masks containing only the 500 highest absolute r-values respectively. Brain regions are sorted into hemispheres (right structures first, left structures next and central structures last). Each hemisphere contains first brainstem regions (Bs), thalamus (Th), cortical regions (Cx), hippocampus (Hc), amygdala (Am), hypothalamus (Hy) and motor output (Mo).

Supplementary Tables

Supplementary Table 1: Differential hierarchical modulation of nociceptive brain states and behavior by CEl PKCδ$^+$ and SST$^+$ neurons.

Fusing anatomical and functional connectivity (Figure 3**a**,**b**) of the central amygdala circuitry primary interactions via direct synaptic connectivity (↔) through bottom-up and top-down mechanisms to differentially modulate nociception. Secondary modulation through indirect connectivity (≈) also contributes.

**Processing hierarchies**	**CEl PKCδ$^+$ effects**	**CEl SST$^+$ effects**
Cortical top-down control in cxCg, cxS1		(≈) Reduced modulation of cortical nociceptive representation in cxS1 by cxCg
Primary representation of nociceptive signals in cxS1, mTh, lTh, BFB	(↔) Uncoupling of BFB and thalamocortical nociceptive sensory representation in mTh, lTh and cxS1 by direct bottom-up interaction with BFB	
Control of aversive behavioral states in Hc, Hy, Am, PAG	(↔) Uncoupling of aversive behavioral states in Hc, Hy, Am and PAG from thalamocortical nociceptive representation by direct bottom-up interaction with Hy	
Relay of sensory nociceptive information in cxM, Hc, Bs	(≈) Increased modulation of nociceptive Bs signals by cxM and Hc	(↔) Reduced antinociceptive modulation of nociceptive Bs signals by direct top-down interaction from Hc

Am (amygdala), BFB (basal forebrain including septum, diagonal band of broca, nucleus accumbens, pallidum), Bs (brainstem), cxCg (cingulate cortex), cxM (motor cortex), cxS1 (prim. somatosensory cortex), Hc (hippocampus), Hy (hypothalamus), lTh (lateral thalamus), mTh (medial thalamus), PAG (periaqueductal gray).

References

1. Paxinos, G. & Franklin, K. *The Mouse Brain in Stereotaxic Coordinates, Compact*. (2008).
2. Haubensak, W. *et al.* Genetic dissection of an amygdala microcircuit that gates conditioned fear. *Nature* **468**, 270–276 (2010).
3. McCullough, K. M., Morrison, F. G., Hartmann, J., Carlezon, W. A. & Ressler, K. J. Quantified coexpression analysis of central amygdala subpopulations. *eNeuro* **5**, (2018) doi:10.1523/ENEURO.0010-18.2018.
4. Kim, J., Zhang, X., Muralidhar, S., LeBlanc, S. A. S. A. & Tonegawa, S. Basolateral to Central Amygdala Neural Circuits for Appetitive Behaviors. *Neuron* **93**, 1464-1479 e5 (2017).
5. Griessner, J. *et al.* Central amygdala circuit dynamics underlying the benzodiazepine anxiolytic effect. *Mol. Psychiatry* (2018) doi:10.1038/s41380-018-0310-3.
6. Allen Institute for Brain Science. Allen Mouse Brain Connectivity Atlas. http://connectivity.brain-map.org/ (2011).
7. Taniguchi, H. *et al.* A Resource of Cre Driver Lines for Genetic Targeting of GABAergic Neurons in Cerebral Cortex. *Neuron* **71**, 995–1013 (2011).
