# Supplementary material for: Central amygdala circuitry modulates nociceptive processing through differential hierarchical interaction with affective network dynamics
Source: Commun Biol. 2021 Jun 14;4:732. doi: 10.1038/s42003-021-02262-3 (PMC8203648; doi:10.1038/s42003-021-02262-3)
Supplement: Supplementary file 3 — Description of Additional Supplementary Files [file 42003_2021_2262_MOESM3_ESM.pdf]

**Description of Additional Supplementary Files**

File Name: Supplementary Data 1
Description: Supplementary data for Figure 2b atp plots.

File Name: Supplementary Data 2
Description: Supplementary data for Figure 3 network analysis.

File Name: Supplementary Data 3
Description: Supplementary data for gene expression analysis.
