# Supplementary material for: Central amygdala circuitry modulates nociceptive processing through differential hierarchical interaction with affective network dynamics
Source: Commun Biol. 2021 Jun 14;4:732. doi: 10.1038/s42003-021-02262-3 (PMC8203648; doi:10.1038/s42003-021-02262-3)
Supplement: Supplementary file 7 — Reporting Summary [file 42003_2021_2262_MOESM7_ESM.pdf]

nature research

Corresponding author(s): Wulf Haubensak
Andreas Hess

Last updated by author(s): May 10, 2021

Reporting Summary

Nature Research wishes to improve the reproducibility of the work that we publish. This form provides structure for consistency and transparency in reporting. For further information on Nature Research policies, see our Editorial Policies and the Editorial Policy Checklist.

Statistics

For all statistical analyses, confirm that the following items are present in the figure legend, table legend, main text, or Methods section.

n/a	Confirmed	
☐	☒	The exact sample size (*n*) for each experimental group/condition, given as a discrete number and unit of measurement
☐	☒	A statement on whether measurements were taken from distinct samples or whether the same sample was measured repeatedly
☐	☒	The statistical test(s) used AND whether they are one- or two-sided<br>*Only common tests should be described solely by name; describe more complex techniques in the Methods section.*
☐	☒	A description of all covariates tested
☐	☒	A description of any assumptions or corrections, such as tests of normality and adjustment for multiple comparisons
☐	☒	A full description of the statistical parameters including central tendency (e.g. means) or other basic estimates (e.g. regression coefficient) AND variation (e.g. standard deviation) or associated estimates of uncertainty (e.g. confidence intervals)
☐	☒	For null hypothesis testing, the test statistic (e.g. *F*, *t*, *r*) with confidence intervals, effect sizes, degrees of freedom and *P* value noted<br>*Give P values as exact values whenever suitable.*
☒	☐	For Bayesian analysis, information on the choice of priors and Markov chain Monte Carlo settings
☒	☐	For hierarchical and complex designs, identification of the appropriate level for tests and full reporting of outcomes
☒	☐	Estimates of effect sizes (e.g. Cohen's *d*, Pearson's *r*), indicating how they were calculated

Our web collection on statistics for biologists contains articles on many of the points above.

Software and code

Policy information about availability of computer code

Data collection	Sequencing data: Published GEO: GSE95154.<br>FMRI data: 4.7 T Bruker Biospec 4740 small animal MRT (Bruker BioSpin MRI GmbH, Ettlingen, Germany) operated by ParaVision 5.1<br>Behavioral data: Model 37450 electronic dynamic plantar aesthesiometer; electronically controlled hotplate analgesia apparatus (IITC Life 447 Science, Woodland Hills, USA).
Data analysis	Statistical genetic analyses: Bowtie2 v2.3.4.1; star v2.6.0c; featureCounts; DESeq2 v1.26.0; scytoscape 3.8.1.<br>FMRI data: Brainvoyager QX V 2.8.2.2523 (Brain Innovation, Maastricht, Netherlands); MagnAn (BioCom GbR, Uttenreuth; written in IDL 8.5), Microsoft Excel 2016.<br>Behavioral data: GraphPad Prism version 8.

For manuscripts utilizing custom algorithms or software that are central to the research but not yet described in published literature, software must be made available to editors and reviewers. We strongly encourage code deposition in a community repository (e.g. GitHub). See the Nature Research guidelines for submitting code & software for further information.

Data

Policy information about availability of data

All manuscripts must include a data availability statement. This statement should provide the following information, where applicable:

- Accession codes, unique identifiers, or web links for publicly available datasets
- A list of figures that have associated raw data
- A description of any restrictions on data availability

Fig. 1: Sequencing data, published at GEO: GSE95154; Figs. 2, 3: Raw fMRI data is available upon request; Extended Data Fig. 1: Sequencing data, published at GEO:

GSE95154; Extended Data Fig. 3: Raw histological data is available upon request; Extended Data Figs. 4, 5, 6, 7: Raw fMRI data is available upon request; Extended Data Fig. 6: Raw behavioral data is available upon request.

Field-specific reporting

Please select the one below that is the best fit for your research. If you are not sure, read the appropriate sections before making your selection.

☒ Life sciences ☐ Behavioural & social sciences ☐ Ecological, evolutionary & environmental sciences

For a reference copy of the document with all sections, see nature.com/documents/nr-reporting-summary-flat.pdf

Life sciences study design

All studies must disclose on these points even when the disclosure is negative.

Sample size

Behavior
Samples sizes (Supplementary Table 2) were pre-determined from previous behavioral experiments (Griessner et al. 2018).
For the behavioral experiments, 30 PKCδ::Cre and 16 SST::Cre were injected with M3- or 101 green fluorescent protein (GFP)-expressing virus (Table 1). As responsiveness and variance were comparable between both GFP groups (Extended Data Fig. 6), animals were pooled to enhance statistical power.

OfMRI
Samples sizes (Supplementary Table 2) were pre-determined from previous optogenetic fMRI experiments (Griessner et al. 2018).

Griessner, Johannes, Manuel Pasieka, Vincent Böhm, Florian Grössl, Joanna Kaczanowska, Pinelopi Pliota, Dominic Kargl, Barbara Werner, Nadia Kaouane, Sandra Strobelt, Silke Kreitz, Andreas Hess, and Wulf Haubensak. 2018. “Central Amygdala Circuit Dynamics Underlying the Benzodiazepine Anxiolytic Effect.” Molecular Psychiatry.

Data exclusions

Behavior:
Specifically, four animals with incorrect viral expression were excluded from the behavioral analysis after histological examination, where the experimenter was not blinded to the assignment of the groups, as viral expression between test- and control-group is different. Successful viral expression was assessed using PKCδ staining for histological control, as PKCδ is expressed in CEl (in amygdala) and not in neighboring areas, so when the viral expression was inside the limits of PKCδ staining area then the injection was considered successful, otherwise the mouse was excluded from the analysis.

OfRMI:
Seven animals were excluded from analysis due to multiple reasons: five animals lost the implanted optogenetic fiber, one animal died before measurement and one had a crippled hind paw that prevented correct and secure fixation of the Peltier heating element.

Replication

The experiments were performed once.

Randomization

Behavior:
Animals from each genotype were randomly assigned to either control or M3/ChR2 groups (Supplementary Table 2).

OfMRI:
Of the 15 animals per mouse strain, five animals were randomly assigned to the control GFP-group and 10 to the ChR2-group (Supplementary Table 3).

Blinding

The experimenter was not blind to surgery, but blinded to the assignment of the behavioral groups. Behavioral scoring was done automatically by the apparatus software.
For fMRI, the analyst was not blinded, but analysis was carried out with all animals taken together in formalized computational workflows to avoid bias effects. Following this work-flow, no animal-specific input to introduce bias was possible.
Histological validation was performed not blinded to group assignment, but blinded to the per animal outcome of behavioral or fMRI experiments.

Reporting for specific materials, systems and methods

We require information from authors about some types of materials, experimental systems and methods used in many studies. Here, indicate whether each material, system or method listed is relevant to your study. If you are not sure if a list item applies to your research, read the appropriate section before selecting a response.

Materials & experimental systems

n/a	Involved in the study
☐	☒ Antibodies
☒	☐ Eukaryotic cell lines
☒	☐ Palaeontology and archaeology
☐	☒ Animals and other organisms
☒	☐ Human research participants
☒	☐ Clinical data
☒	☐ Dual use research of concern

Methods

n/a	Involved in the study
☒	☐ ChIP-seq
☒	☐ Flow cytometry
☐	☒ MRI-based neuroimaging

Antibodies

Antibodies used: Anti-PKCδ (IgG 2b), dilution 1:1000, 610398, BD Biosciences.

Validation: Anti-PKCδ (IgG 2b), dilution 1:1000, 610398, BD Biosciences and previous work (Griessner et al. 2018).

Griessner, Johannes, Manuel Pasieka, Vincent Böhm, Florian Grössl, Joanna Kaczanowska, Pinelopi Pliota, Dominic Kargl, Barbara Werner, Nadia Kaouane, Sandra Strobelt, Silke Kreitz, Andreas Hess, and Wulf Haubensak. 2018. "Central Amygdala Circuit Dynamics Underlying the Benzodiazepine Anxiolytic Effect." Molecular Psychiatry.

Animals and other organisms

Policy information about studies involving animals; ARRIVE guidelines recommended for reporting animal research

Laboratory animals: C57BL/6J or transgenic male mice (Tg(Prkcd-glc-1/CFP,-cre)EH124Gsat MGI:3844446 87 (PKCδ::Cre) or Sst-IRES-Cre knock-in (SST::Cre) (Jackson Laboratory stock no: 028864) 88 backcrossed to C57BL/6J)

Wild animals: N/A

Field-collected samples: N/A

Ethics oversight: All animal procedures were performed in accordance with institutional guidelines and were approved by 350 the respective Austrian and German authorities covered by the license M58/002220/2011/9.

Note that full information on the approval of the study protocol must also be provided in the manuscript.

Magnetic resonance imaging

Experimental design

Design type: Event-related

Design specifications: The 65 min ofMRI session contained 8 stimulus blocks, each consisting of 1) heat stimulus (50 °C for 20 s [15 s ramp, 5 s plateau]), 2) laser stimulus (10 mW and 10 Hz frequency at a wavelength of 473 nm for 20 s), 3) a simultaneous combination of laser and heat followed by 4) again a laser stimulus (3) and 4) same settings as 1) and 2)).
The stimulus interval as well as block interval was always 100 s. Each subject underwent this protocol only once.

Behavioral performance measures: N/A

Acquisition

Imaging type(s): functional

Field strength: 4.7 Tesla

Sequence & imaging parameters: Gradient Echo Planar Imaging (TR = 2000 ms; TEef = 25.3 ms, flip angle 90°, FOV = 15 mm * 15 mm; slice thickness 0.5 mm; matrix size 64*64 voxel, in-plane resolution of 0.234 mm * 0.234 mm, 22 axial slices)

Area of acquisition: The 22 axial slices were covering the brain from Bregma -2.06 mm to 1.42 mm; positioning of the volume according to an anatomical landmark

Diffusion MRI: ☐ Used ☒ Not used

Preprocessing

Preprocessing software	Preprocessing, after discarding the first 2 volumes of the datasets avoiding MR saturation effects, comprised slice scan time correction (ascending interleaved, interpolation method cubic spline), motion correction to eliminate the minimal mouse head movement (registration to first brain volume; trilinear detection and sinc interpolation), spatial (Gaussian smoothing with kernel size of 2 pixel) and temporal smoothing (linear and non-linear high pass filtering, kernel 12s FWHM, FFT 9 cycles) was performed in Brainvoyager QX (Brain Innovation, Maastricht, Netherlands; V 2.8.2.2523).
Normalization	Included and stated in preprocessing (subject-wise high-pass filtering);
Normalization template	study specific template generated by affine registration with 6 degree of freedom (translation x-, y-, and z-axis, rotation z-axis, tilt in z-axis, scale in x-y-axis)
Noise and artifact removal	Included and stated in preprocessing
Volume censoring	N/A

Statistical modeling & inference

Model type and settings	General linear model to calculate statistical coupling to stimulation protocol; 3 separate predictors for 1) heat-only, 2) laser-only, and 3) laser-heat co-stimulation;
Effect(s) tested	Differences in BOLD signal amplitude between ChR2-groups and GFP-controls (one factor repeated measures ANOVA with subsequent Tukey HSD);<br>graph-theoretical analysis: brain region-wise differences in Pearson correlation between the mean BOLD time courses of ChR2-groups and GFP-controls (homoscedastic Student's T-Test);

Specify type of analysis: ☐ Whole brain ☐ ROI-based ☒ Both

Anatomical location(s)	Labeling of 196 brain regions in subject-space using a digital 3D modified Paxinos mouse brain atlas
Statistic type for inference (See Eklund et al. 2016)	GLM was calculated voxel-wise; FDR thresholding of the resulting statistical parametric maps yielded the significantly activated voxels used for a) differences in BOLD signal amplitude, and b) graph-theoretical analysis performed on mean time courses of the labeled brain regions;
Correction	FDR (see above) and Bonferroni

Models & analysis

n/a	Involved in the study
☐	☒ Functional and/or effective connectivity
☐	☒ Graph analysis
☐	☐ Multivariate modeling or predictive analysis

Functional and/or effective connectivity	Pearson correlation
Graph analysis	After removal of the global mean, the Pearson correlation coefficient r was calculated between the full-length average time courses of all 196 brain regions for each animal.<br>This yielded one correlation matrix per subject and predictor, representing the similarity of the time courses across all brain structures.<br>R-values were converted into Fisher-z-values to calculate one mean undirected correlation matrix per group and predictor. The mean adjacency matrices were converted back into r-values, representing the functional connectivity between the time courses. To allow for an optimal topological comparison, matrices had to be limited to contain the same number of connections (500 strongest positive r-values as well as the 500 lowest negative r-values) resulting in the frequently used k-value of 10 for the topological comparison.
Multivariate modeling and predictive analysis	N/A

April 2020
